# Bacteriophage‐Activated DNAzyme Hydrogels Combined with Machine Learning Enable Point‐of‐Use Colorimetric Detection of *Escherichia coli*

**DOI:** 10.1002/adma.202411173

**Published:** 2024-11-26

**Authors:** Hannah Mann, Shadman Khan, Akansha Prasad, Fereshteh Bayat, Jimmy Gu, Kyle Jackson, Yingfu Li, Zeinab Hosseinidoust, Tohid F. Didar, Carlos D. M. Filipe

**Affiliations:** ^1^ Department of Chemical Engineering McMaster University 1280 Main Street West Hamilton ON L8S 4L8 Canada; ^2^ School of Biomedical Engineering McMaster University 1280 Main Street West Hamilton ON L8S 4L8 Canada; ^3^ Department of Biochemistry and Biomedical Sciences McMaster University 1280 Main Street West Hamilton ON L8S 4L8 Canada; ^4^ Department of Mechanical Engineering McMaster University Hamilton ON L8S 4L7 Canada

**Keywords:** clinical diagnostics, colorimetric sensor, contamination sensing, DNAzyme, *E. coli* detection, hydrogel sensor, machine learning

## Abstract

Developing cost‐effective, consumer‐accessible platforms for point‐of‐use environmental and clinical pathogen testing is a priority, to reduce reliance on laborious, time‐consuming culturing approaches. Unfortunately, a system offering ultrasensitive detection capabilities in a form that requires little auxiliary equipment or training has remained elusive. Here, a colorimetric DNAzyme‐crosslinked hydrogel sensor is presented. In the presence of a target pathogen, DNAzyme cleavage results in hydrogel dissolution, yielding the release of entrapped gold nanoparticles in a manner visible to the naked eye. Recognizing that *Escherichia coli* holds high relevance within both environmental and clinical environments, an *E. coli*‐responsive DNAzyme is incorporated into this platform. Through the optimization of the hydrogel polymerization process and the discovery of bacteriophage‐induced DNAzyme signal amplification, 10^1^ CFU mL^−1^ *E. coli* is detected within real‐world lake water samples. Subsequent pairing with an artificial intelligence model removed ambiguity in sensor readout, offering 96% true positive and 100% true negative accuracy. Finally, high sensor specificity and stability results supported clinical use, where 100% of urine samples collected from patients with *E. coli* urinary tract infections are accurately identified. No false positives are observed when testing healthy samples. Ultimately, this platform stands to significantly improve population health by substantially increasing pathogen testing accessibility.

## Graphical abstract

A colorimetric hydrogel sensing platform for the detection of pathogens in water and clinical samples is described. The polyacrylamide hydrogel is held together with pathogen‐responsive DNAzyme‐substrate cross‐linking and has demonstrated high sensitivity for detecting *E. coli* in lake water and urine. The bacteriophage‐mediated signal amplification effect outlined in the design has potential applications for future biosensor development.

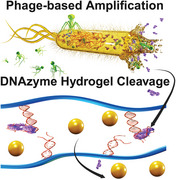

## 1 Introduction

With the emergence of antibiotic resistance, limiting pathogenic exposure has become a growing priority. Currently, one of the most defined mechanisms for such exposure involves the consumption of unsafe drinking water, with an estimated 1.8 billion people relying on water sources that are prone to fecal contamination.^[^ ^1^ ^]^ A prevalent issue within low‐income and rural communities, inadequate treatment of such water supplies prior to consumption yields a significant risk of disease.^[^ ^2^ ^]^ To this end, effective water monitoring is key, wherein *Escherichia coli* (*E. coli*) is often used as an indicator of water safety.^[^ ^3^, ^4^ ^]^ While not always pathogenic in nature, its prevalence in the digestive tract of humans and animals makes it an effective marker of fecal contamination. When infection does occur – irrespective of the source, rapid diagnosis is particularly important toward ensuring optimal patient outcomes. Here, *E. coli* is one of the leading causes of disease, accounting for an estimated 950K annual deaths globally.^[^ ^5^ ^]^ Once again, severe ailments in this space disproportionally impact rural and low‐income communities, due to a lack of effective testing resources. There is an established need for effective *E. coli* detection for both environmental monitoring and clinical diagnosis, particularly in low‐income and remote communities.

While various approaches for bacterial detection are currently employed, conventional methods have requirements that limit their widespread applicability. For example, bacterial culturing, which remains the gold‐standard approach for *E. coli* detection, is time‐consuming, relies on trained personnel, and requires specialized laboratory equipment.^[^ ^6^, ^7^ ^]^ In an effort to alleviate these drawbacks, many on‐site detection platforms have been proposed in the literature.^[^ ^8^ ^]^ This includes electrochemical,^[^ ^9^ ^]^ enzymatic,^[^ ^10^, ^11^ ^]^ aptamer,^[^ ^12^, ^13^ ^]^ and antibody‐based methods.^[^ ^14^, ^15^ ^]^ Despite promising offerings, no platform has offered high sensitivity and specificity toward *E. coli* in a form factor that is easy to distribute, simple to use, requires minimal equipment, works with both urine and real‐world water samples, and is faster than conventional approaches.

Given these requirements, bacteria‐responsive RNA‐cleaving DNAzymes are particularly promising for such on‐site applications.^[^ ^6^, ^16^, ^17^ ^]^ These molecules generally consist of an enzymatic strand hybridized to a substrate strand. Once a target binds to the enzymatic strand, its catalytic activity is activated, yielding cleavage at a ribonucleotide site located within the substrate strand. Owing to their ability to function as stable, standalone detection molecules, these molecules yield sensitive and specific biosensors that require limited handling and processing.^[^ ^18^ ^]^ This has inspired the development of several DNAzyme‐based fluorescence biosensors for bacterial detection from our group, including for *E. coli*.^[^ ^19^, ^20^, ^21^, ^22^ ^]^ However in order for a system not to be reliant on specialized equipment like fluorescence imaging devices, it must offer colorimetric signal transduction to enable qualitative sensor assessment with the naked eye.^[^ ^18^ ^]^ To this end, previous works have detailed DNAzyme crosslinked hydrogels for the colorimetric detection of heavy metals using metal ion‐specific DNAzymes.^[^ ^23^, ^24^, ^25^ ^]^ Briefly, these hydrogels involve the entrapment of colored agents within a polymer chain network that is held together by target‐responsive DNAzymes.^[^ ^26^ ^]^ Gel dissolution in the presence of the target yields visible color release.

Importantly, while metal ion targets can readily diffuse through such hydrogel matrices to activate gel dissolution, bacterial targets are much larger in size. Fortunately, bacteria‐responsive DNAzymes are not activated by whole cells, but rather by highly specific entities (i.e., proteins) secreted by the target cell. Yet, recognizing the extensive DNAzyme crosslinking that holds such a gel together, effective bacterial detection would require a significant concentration of the cleavage‐inducing entity in the test solution. Introducing an equipment‐free approach for bacterial lysis thus represents a promising approach toward signal amplification. That being said, both environmental and clinical samples are rich in microbes, meaning that targeted lysis is essential to limit the unwanted presence of non‐specific entities in the microscale hydrogel matrix.

Herein, we present a DNAzyme‐based colorimetric hydrogel biosensor for the on‐site detection of *E. coli* in complex samples. The biosensor consists of a polyacrylamide hydrogel crosslinked with a novel *E. coli*‐responsive DNAzyme construct that is activated by an *E. coli*‐specific protein (ECP1). The hydrogel matrix is embedded with gold nanoparticles (AuNPs), that offer colorimetric transduction in response to ECP1‐induced DNAzyme cleavage. The biosensor also incorporates Bacteriophage T7 – an *E. coli*‐infecting phage with high lytic activity,^[^ ^27^, ^28^, ^29^ ^]^ to specifically maximize ECP1 availability, yielding signal amplification. Notably, this is the first report of bacteriophage‐induced amplification of DNAzyme signaling. The resultant sensor offered highly specific and sensitive detection of *E. coli*, with concentrations as low as 10^1^ CFU mL^−1^ inducing color shifts visible to the naked eye within 18 h. The sensor also offered excellent stability in response to various environmental stressors, effectively maintaining its detection capabilities. When tested with real‐world environmental water samples and clinical urine samples, application‐relevant detection performance was observed. Finally, an artificial intelligence (AI) network was developed to automatically classify gels based on the degree of color shift, to improve system accessibility and remove user ambiguity. The network effectively classified tested biosensors as either contaminated or uncontaminated with a 96% true positive accuracy.

## 2 Results and Discussion

### 2.1 Fabrication and Design of the *E. coli*‐Responsive Hydrogel Biosensor

**Figure 1:**
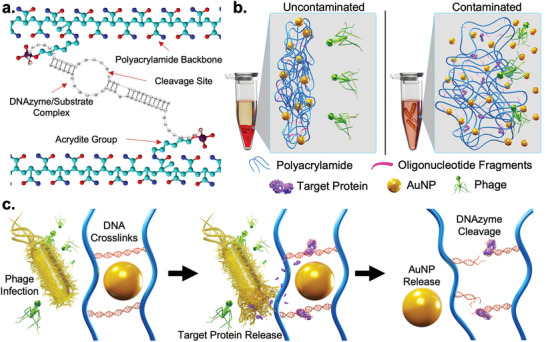
Schematics of colorimetric *E. coli* detecting platform. a) Chemical structure of the polymeric matrix showing an acrydite DNAzyme/substrate crosslink. b) Macro and microscale schematics of the hydrogel matrix performance under contaminated or uncontaminated conditions. c) Intended functionality of the sensor under contaminated sample conditions.

The developed hydrogel biosensor is composed entirely of polyacrylamide chains and oligonucleotides, wherein the polymer chains are held together by acrydite‐modified, *trans* conformation *E. coli*‐responsive DNAzymes (**Figure** **1**a; Figures S1 and S2, Table S1, Supporting Information). This acrydite modification enables the copolymerization of the enzymatic and substrate oligonucleotides with acrylamide monomers during chain polymerization. The two sequences are individually copolymerized and then mixed together to induce hybridization‐based DNAzyme crosslinking. AuNPs introduced during this step are entrapped within the gel matrix. As this study sought to develop a biosensor that could be used on‐site by untrained users with no access to specialized auxiliary equipment, the hydrogel biosensor was embedded within a tube (Figure 1b). To this, the user can simply add their test sample, alongside pre‐packaged reagents. When a test sample is added to the tube, it slowly permeates through the hydrogel sensing matrix, with a significant excess of the added solution overlaying the gel. If *E. coli* is present in the tube, free‐flowing ECP1 interacts with the DNAzyme, inducing a cleavage reaction that results in gel dissolution‐driven AuNP release that is visible to the naked eye (Figure 1c; Figure S3, Supporting Information).

### 2.2 Optimization and Characterization of Polymer and Hydrogel Detection Mechanism

**Figure 2:**
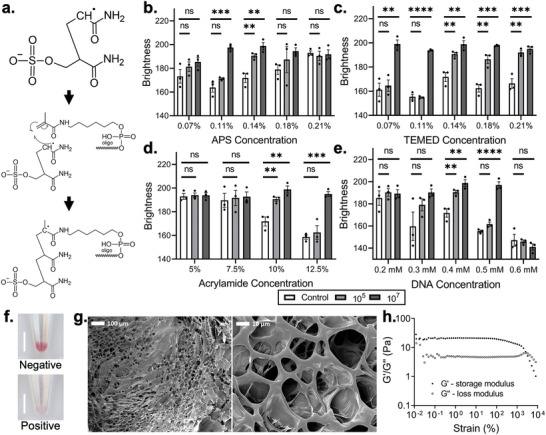
Characterization and optimization of the hydrogel detection system. a) Chemical diagram showing how an acrydite modified oligonucleotide fragment is added to a polyacrylamide chain during FRP. b–e) Sensitivity testing of gels polymerized using different concentrations of APS, TEMED, acrylamide monomer, and acrydite modified DNA, respectively. Sensitivity was evaluated at 10^5^ and 10^7^ CFU mL^−1^ of *E. coli*. Sample size *n* = 3 for each test category. f) Optical images of the colorimetric detection platform, showing negative and positive results. Scale bars represent 5 mm. g) SEM images of freeze‐dried hydrogel structure at 100 × and 1000 × magnification. h) Rheology result of a strain sweep performed at a fixed frequency of 1 Hz at 25 °C. Asterisks represent “ns” (*p* > 0.05), ^*^ (*p* ≤ 0.05), ^**^ (*p* ≤ 0.01), ^***^ (*p* ≤ 0.001), and ^****^ (*p* ≤ 0.0001) from ANOVA with Dunnett's multiple comparisons test. All error bars represent the standard error of the mean.

The polymer components of the developed hydrogel detection platform were synthesized using free radical polymerization (FRP) (**Figure** **2**a). As mentioned above, two identical polymerizations were completed, with one containing the catalytic oligonucleotide strand and the other containing the substrate strand. The conditions under which an FRP reaction is performed dictate the characteristics of the resultant polymer products. When subsequently used to produce hydrogels, these characteristics influence the mechanical and chemical properties of the gel. In particular, the concentrations of the initiator, catalyst, and monomer dictate chain length and determine the viscosity of the resultant hydrogel.^[^ ^30^ ^]^ With regards to sensing, this impacts the accessibility of the DNAzyme crosslinking sites, as well as the hydrogel's ability to effectively entrap colored agents. On the other hand, acrydite‐modified DNA concentration determines the cross‐linking density – a sensing parameter that needs to balance high hydrogel stability in the absence of the target, with efficient target‐induced breakage.

To maximize the performance of our *E. coli*‐responsive hydrogel biosensor, the concentrations of each of these components (Figure S4, Supporting Information) were optimized. Specifically, hydrogels produced with varying reagent compositions were tested under both *E. coli*‐positive (10^7^ CFU mL^−1^, 10^5^ CFU mL^−1^) and *E. coli*‐negative conditions. Ammonium persulfate (APS) and *N,N,N′,N′*‐Tetramethyl ethylenediamine (TEMED) were used as the initiator and catalyst, respectively, with both being evaluated at concentrations between 0.07% to 0.21% (Figure 2b,c). When an APS concentration of 0.07% was employed, the fabricated hydrogels were not stable, as insufficient initiation yielded limited chain formation. While stable hydrogels were produced at an APS concentration of 0.11%, they did not respond to the presence of *E. coli* at a concentration of 10^3^ CFU mL^−1^. Contrarily, an APS concentration of 0.14% yielded an effective 10^3^ CFU mL^−1^ *E. coli* detection. It is hypothesized that the lower frequency of initiation events that occur at an APS concentration of 0.11% yield longer polymer chains that impede ECP1 diffusion into the hydrogel matrix. When APS concentration was increased to 0.18% and 0.21%, the resultant hydrogels were mechanically weak, as excess initiation yielded short polymer chains unable to form a stable bulk material. This results in a significant risk of false positives, as AuNPs are not effectively entrapped within the polymer matrix. An APS concentration of 0.14% was thus considered optimal. With regards to TEMED, concentrations of 0.14%, 0.18%, and 0.21% all yielded hydrogels that offered optimal performance across the three test conditions. That being said, hydrogels produced using 0.14% TEMED offered the greatest mechanical stability, by drawing a balance between monomer accessibility for initiation and chain elongation rate. This concentration was thus selected.

In relation to the acrylamide monomer, concentrations of 5%, 7.5%, 10%, and 12.5% w/v were evaluated (Figure 2d). Hydrogels could not be produced using monomer concentrations exceeding 12.5%, as the FRP products were too viscous for processing. At monomer concentrations of 5% and 7.5%, a solid hydrogel did not form, as sufficient chain length was likely not achieved. An increase in concentration to 10% resolved this issue, offering effective detection performance across all three test conditions. Further increasing the monomer concentration to 12.5% still yielded stable hydrogels, but 10^3^ CFU mL^−1^ *E. coli* was not detected – likely due to increased DNAzyme inaccessibility caused by longer polymer chains. A 10% acrylamide monomer concentration was thus used within subsequent synthesis.

Finally, the concentration of acrydite‐modified DNA was evaluated at concentrations between 0.2 mM and 0.6 mM (Figure 2e). At concentrations of 0.2 and 0.3 mm, fabricated hydrogels lacked stability, yielding unreliable detection. Specifically, the reduced crosslinking density within these hydrogels caused spontaneous breakage in the absence of *E. coli*, resulting in false positive signaling. Stability was afforded with a DNA concentration of 0.4 mM, wherein effective detection was also observed. However, using DNA concentrations of 0.5 and 0.6 mm yielded hydrogels that were not responsive to 10^3^ CFU mL^−1^ *E. coli*, as their increased crosslinking density requires higher ECP1 target availability for breakage. The 0.4 mm concentration was thus selected.

Collectively, these optimized parameters produced hydrogels with an appropriate trade‐off between physical stability and detection performance (Table S2, Supporting Information). The resulting signal was visible to the naked eye (Figure 2f). These optimized hydrogels were visualized using scanning electron microscopy (SEM) and rheology. SEM was performed after freeze drying to preserve the hydrogel structure, wherein pores with diameters between 10 and 20 µm were observed (Figure 2g; Figure S5, Supporting Information). G’ (storage modulus) was greater than G’’ (loss modulus) for the hydrogel rheology tests until fluid‐like behavior at strains exceeding 1000% (Figure 2h). The same was not true for the individual polymer components before the enzymatic and substrate strands were mixed (Figures S6 and S7, Supporting Information), showing the gelation impact of hybridization.

### 2.3 Bacteriophage and Machine Learning Integration for Environmental Sample Testing

**Figure 3:**
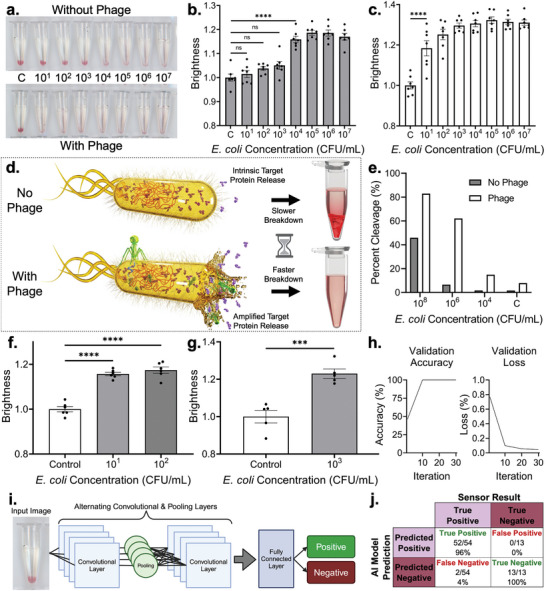
Bacteriophage and machine learning integration, applied towards environmental sample testing. a) Optical images of colorimetric detection of *E. coli* after an 18 h incubation at 40 °C, with and without the addition of T7 phage after 6 h. Numbers represent CFU mL^−1^. b–c) Quantified results for the experiments pictured in part A. Each dataset's brightness values have been normalized to their respective controls. Panel B shows results without phage, panel C shows results with phage introduced after 6 h. Sample size *n* = 7 for each concentration. d) Schematic of the proposed mechanism of phage increasing DNAzyme cleavage. T7 bacteriophage attaching to *E. coli*, injecting its DNA, and inducing bacterial cell lysis, resulting in increased amounts of bacterial proteins being released into the extracellular environment. e) Electrophoresis gel results quantifying the percentage of DNAzyme that gets cleaved by different concentrations of *E. coli*, with and without phage. Measurements were taken after 24 h. f) Detection performance of the platform for 10^1^ and 10^2^ CFU mL^−1^ *E. coli* lake water samples. Sample size n = 6 for each concentration. g) Detection performance of the platform for 10^3^ CFU mL^−1^ *E. coli* cistern water samples. *n* = 5 for each concentration. h) Validation accuracy and loss plots from the CNN trained on the colorimetric gel images. i) Schematic of AI analysis of gel images using a CNN. j) Confusion matrix of the AI model output when assessed with a testing dataset of 67 images. Asterisks represent “ns” (*p* > 0.05), ^*^ (*p* ≤ 0.05), ^**^ (*p* ≤ 0.01), ^***^ (*p* ≤ 0.001), and ^****^ (*p* ≤ 0.0001) from ANOVA with Dunnett's multiple comparisons test or two‐tailed *t*‐test (panel g). All error bars represent the standard error of the mean.

With optimal polymerization parameters identified, the sensitivity of the sensor was tested through incubation with water samples contaminated with 10^1^ to 10^7^ CFU mL^−1^ *E. coli* (**Figure** **3**a). The limit of detection (LOD) was qualitatively and quantitatively determined to be 10^4^ CFU mL^−1^ (Figure 3b). While promising, this level of performance is not sufficient for many real‐world applications. While culturing samples prior to testing would overcome this issue, such a step would both increase the assay time and limit on‐site, untrained sensor use. We hypothesized that a similar improvement in LOD could be enacted through the use of *E. coli*‐specific lytic T7 bacteriophage.^[^ ^31^, ^32^, ^33^ ^]^ Here, these agents would lyse and thus release all DNAzyme‐activating proteins present within intracellular environments, significantly increasing cleavage activity without increasing assay time. This proposed cascade was explored using T7 bacteriophage, wherein the sensor's LOD was improved by several orders of magnitude to 10^1^ CFU mL^−1^ (Figure 3c), as confirmed by qualitative visual inspection and quantitative analysis (Figure 3a; Figure S8, Supporting Information). Given that qualitative detection via visual inspection offered such high sensitivity, it can be noted that this sensor does not require any equipment for qualitative ultrasensitive sample analysis. This sensor thus has considerable potential for on‐site testing of environmental and clinical samples. The bacteriophage‐mediated sensing mechanism is visualized in Figure 3d.

To further validate the proposed bacteriophage amplification mechanism, direct cleavage tests were performed on non‐crosslinked DNAzyme samples with and without T7 bacteriophage (Figure 3e). Here, cleavage rate was quantified through gel electrophoresis, wherein bands formed by cleavage fragments provided a measure of percent cleavage in a given sample. Here, the samples with bacteriophage achieved a percent cleavage of 82.92% at 10^8^ CFU mL^−1^ *E. coli*. By contrast, 10^8^ CFU mL^−1^ *E. coli* samples without bacteriophage only had a percentage cleaved of 45.86%.

To assess the suitability of this platform for real‐world environmental testing, lake water, and cistern water – both prone to microbial contamination,^[^ ^3^, ^4^, ^34^ ^]^ were spiked with low concentrations of *E. coli* and tested (Figure 3f,g; Table S3, Supporting Information). *E. coli* was successfully detected in both lake water and cistern water using this platform, at concentrations as low as 10^1^ and 10^3^ CFU mL^−1^, respectively (Figure S9, Supporting Information).

To further automate and increase the ease of use of this platform, a convolutional neural network (CNN) AI model was trained from the optical images (Figure 3h,i). The strong image analysis capabilities of this network make it well‐suited for this application.^[^ ^35^ ^]^ Images used to create the model were split randomly into training and validation data. After every 10 iterations of training the model with the training data, it was assessed with the rest of the images for validation. This resulted in a final validation accuracy of 100% for the model. Training accuracy and loss plots are shown in Figure S10 (Supporting Information). The model was then assessed using a separate testing dataset that included images of sensors exposed to different concentrations of *E. coli*, ranging from 10^1^ to 10^7^ CFU mL^−1^. This final assessment resulted in a true positive rate of 96% and a true negative rate of 100%, as shown in a confusion matrix (Figure 3j). By enabling automated sensor readout using a simple optical image, this AI model effectively eliminates ambiguity surrounding colorimetric readout, further increasing the accessibility and ease of use of the developed sensor.

### 2.4 Sensor Specificity, Stability, and Performance Against Clinical Urine Samples

**Figure 4:**
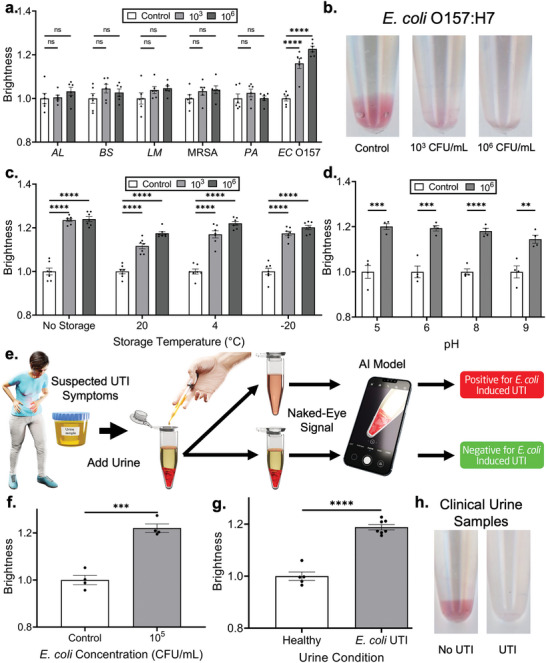
Sensor specificity, stability, and performance against clinical urine samples. a) Detection specificity of the platform when tested with various other species of bacteria at concentrations of 10^3^ and 10^6^ CFU mL^−1^. Bacterial species are as follows: AL – *Acinetobacter lwoffii*, BS – *Bacillus subtilis*, LM – *Listeria monocytogenes*, PA – *Pseudomonas aeruginosa*, and MRSA – methicillin‐resistant *Staphylococcus aureus*. Sample size *n* = 6 for each concentration. b) Optical images showing naked‐eye detection of *E. coli* 0157:H7 at concentrations of 10^3^ and 10^6^ CFU mL^−1^. Image taken after 18 h at 40 °C. c) Detection stability after storage for two weeks under different temperature conditions. *n* = 7 at each condition. d) Detection performance of the platform for 10^6^ CFU mL^−1^ *E. coli* with water samples of differing pH values. *n* = 4 at each condition. e) Schematic demonstrating the potential use of this sensing platform for at‐home UTI detection. f) Detection performance of the platform on healthy urine samples spiked with 10^5^ CFU mL^−1^ of *E. coli*. *n* = 4 at each condition. g) Detection performance of the platform on both healthy urine samples and *E. coli* contaminated urine samples from patients with UTIs. *n* = 5 healthy samples and 7 infected samples. h) Optical images showing naked‐eye detection of *E. coli‐*contaminated clinical urine samples. Asterisks represent “ns” (*p* > 0.05), ^*^ (*p* ≤ 0.05), ^**^ (*p* ≤ 0.01), ^***^ (*p* ≤ 0.001), and ^****^ (*p* ≤ 0.0001) from ANOVA with Dunnett's multiple comparisons test or two‐tailed *t*‐test (panels d, f, and g). All error bars represent the standard error of the mean.

Next, we sought to test the specificity of the developed sensor. Here, *E. coli* O157:H7 was used as a positive test sample, while *Acinetobacter lwoffii*, *Bacillus subtilis*, *Listeria monocytogenes*, *Pseudomonas aeruginosa*, and methicillin‐resistant *Staphylococcus aureus* were used as non‐specific test samples (**Figure** **4**a,b). 10^3^ and 10^6^ CFU mL^−1^ bacterial suspensions were employed for each bacterial condition. While a statistically significant signal was observed for both concentrations of *E. coli* O157:H7, the same was not true for the other bacterial species. This affirms that the *trans* confirmation of the EC1 DNAzyme employed in this work maintains the high degree of specificity offered by its previously reported *cis* counterpart.^[^ ^6^, ^36^ ^]^ The retainment of *E. coli* O157:H7‐responsive activity is particularly important, as detection of this pathogen ensures high‐value applicability within various environmental testing circumstances.^[^ ^37^ ^]^

We then assessed the stability of the developed sensor within diverse long‐term storage conditions. Sensors were stored at temperatures of −20, 4, and 20 °C for two weeks and then tested using uncontaminated, 10^3^ CFU mL^−1^ *E. coli*, and 10^6^ CFU mL^−1^ *E. coli* test samples (Figure 4c). Detection performance was compared to that of sensors tested immediately after fabrication. While sensors stored at −20 °C most effectively maintained colorimetric signal intensity in terms of visual appearance, sensors from all storage conditions offered accurate *E. coli* detection. Storage for 8 days at the higher temperature of 30 °C, as well as long‐term gel storage for 5 months at −20 °C also resulted in successful detection at 10^3^ CFU mL^−1^ and 10^6^ CFU mL^−1^ *E. coli* (Figures S11 and S12, Supporting Information). These results further supported the commercial feasibility of the developed sensor.

Next, to evaluate the viability of the developed sensor for the analysis of acidic and basic test samples, the impact of test sample pH on sensor performance was assessed. Water samples with pH levels ranging from 5 to 9 were used to test sensors in both an uncontaminated state and a contaminated state, wherein *E. coli* loads of 10^3^ and 10^6^ CFU mL^−1^ were employed (Figure 4d). The sensor retained its ability to accurately report both the absence and presence of *E. coli* under all tested concentrations. Such pronounced stability across a wide pH range suggests that the developed platform may be suitable for the evaluation of a wide range of test sample types. In particular, these results offered support for clinical sample analysis.

To comprehensively evaluate such a premise, proof‐of‐concept testing was performed using urine samples, where effective *E. coli* monitoring offers significant value toward UTI diagnosis (Figure 4e).^[^ ^38^, ^39^ ^]^ To account for any non‐specific urine‐induced signals, healthy urine was used as a negative control for *E. coli*‐spiked urine samples. Successful detection of the *E. coli* infection was observed, wherein all test samples offered a significantly higher signal than all control samples (Figure 4f). Finally, healthy and infected clinical urine samples collected from local healthcare facilities were tested using the developed sensor to account for any differences in real‐world infection. Here, seven *E. coli*‐infected samples and five *E. coli*‐unrelated samples were tested (Figure 4g,h; Figure S13 and Table S4, Supporting Information). Accurate assessment was enacted with all patient samples, with qualitative detection interpretable to the naked eye (Figure 4h).

## 3 Conclusion

We have created an ultrasensitive hydrogel detection platform that can be applied to water and urine testing. Due to its colorimetric signal that can be qualitatively interpreted with the naked eye and its pH and temperature stability, this sensor is well suited to real‐world on‐site testing of a variety of potentially contaminated water sources as well as for at‐home UTI testing. The CNN AI model that has been developed enables the automatic classification of samples into the categories of contaminated or uncontaminated, based only on optical images. By using T7 bacteriophage for lysis, we have been able to improve the sensitivity of the developed sensor by several orders of magnitude. The phage‐induced improvement to sensor performance reported in this work is notable even separate to this specific platform. This observation potentially has wide‐ranging applicability to other DNAzyme‐based bacterial sensing platforms, where phage may also be useful as an equipment‐free method of inducing bacterial lysis for signal amplification. Comprehensive assessments of such phage‐DNAzyme interactions represent a key area of study for future work. Subsequent works should also explore crosslinking the developed hydrogel sensors with DNAzymes specific to various bacterial targets, to increase the use case of the developed platform. Ultimately, this *E. coli* sensing platform has immediate relevance both for real‐world water and urine testing applications, while also providing a foundation for further research into colorimetric bacteria‐detecting hydrogels and phage‐amplified sensing.

## 4 Experimental Section

### Materials

A total of 0.2 mL clear flat cap PCR tubes were acquired from Diamed Lab Supplies Inc (Ontario, Canada). Ammonium persulfate (APS), *N*,*N*,*N*′,*N*′‐Tetramethyl ethylenediamine (TEMED), 40% acrylamide monomer solution, 1.0 m MgCl_2_ buffer, 30% gold (III) chloride solution, trisodium citrate dihydrate, and bovine serum albumin (BSA) were purchased from Millipore Sigma (Ontario, Canada). Bacto yeast extract was purchased from ThermoFisher Scientific (Ontario, Canada). Acrydite modified oligonucleotides were ordered from Integrated DNA Technologies (IDT) (Iowa, USA).

### Bacterial Preparation

*E. coli* K12, *E. coli* O157:H7, *A. lwoffii*, *B. subtilis*, *L. monocytogenes*, *P. aeruginosa*, and methicillin‐resistant *S. aureus* were each cultured from glycerol stocks in a shaking incubator for 18 h at 37 °C and 180 RPM in suitable media. The culture solutions were then centrifuged at 7000 RCF for 15 min at 4 °C, and the resulting bacterial pellet was resuspended in DI water.

### Nanoparticle Synthesis and BSA Coating

A modified version of the procedure described by Grabar et al. was used.^[^ ^40^ ^]^ To synthesize the gold nanoparticles, 200 uL of gold chloride solution was added to 300 mL of DI water in an Erlenmeyer flask with a magnetic stir bar. This solution was stirred at 1200 RPM and heated to 100 °C, monitored with a temperature probe. A separate solution of 0.5 g trisodium citrate dihydrate in 30 mL of DI water was then added to the flask, with mixing continuing for 10 min. The Erlenmeyer flask was removed from heat and placed in an ice bath, with the solution still being stirred at 1200 RPM for an additional 15 min. To coat the nanoparticles in BSA, 15 mL of the nanoparticle solution was combined with 5 mL of 2% BSA and stirred for 18 h at 300 RPM. The mixture was then centrifuged at 14 000 RPM for 15 min at 4 °C, at which point the supernatant was removed and the particles were resuspended in DI water.

### Phage Propagation and Filtering

T7 phage was propagated using a host, *Escherichia coli* strain K12 BL21 (Sigma‐Aldrich, CMC001). Briefly, a pre‐culture of *E. coli* in LB‐Miller broth was cultured overnight in a shaking incubator (180 RPM at 37 °C). The next day, using fresh LB‐Miller broth, a 1:200 subculture was prepared and allowed to grow until an OD_600_ _nm_ of 0.6 was reached, upon which 10 µL of T7 phage (≈10^10^ PFU mL^−1^) was added. 50 µL of 1 m CaCl_2_ solution was added to the subculture to assist phage infectivity. The T7‐subculture solution was incubated in a shaking incubator (180 RPM at 37 °C) for 6 h. The culture was then centrifuged at 7000 RCF for 15 min. The bacteria pellets were discarded, and the phage‐containing supernatant was retained and filtered through a 0.2 µm filter (Fisher Scientific, 13 100 106) to remove residual bacteria. Filtered supernatant was stored at 4 °C.

### Double Overlay Phage Titer Assay

T7 phage was quantified using the agar overlay technique. Briefly, 200 µL of E. coli K‐12 BL21 and 100 µL of diluted T7 phage solution were added to liquefied LB‐Miller Soft Agar (2.5% LB; 0.6% Agar), vortexed, and poured on top of LB‐Miller Agar (2.5% LB; 1.5% Agar) plates (Fisher Scientific, Sterile 100 mm × 15 mm Polystyrene Petri Dishes, FB0875712). Plates were placed in a stationary incubator set at 37 °C for 6 h and then incubated at room temperature for 12 h. The number of resulting plaques was counted, and a total concentration of 10^9^ PFU mL^−1^ was determined using the following equation:

(1) $$\mathrm{Concentration}=\frac{\#\;\mathrm{of}\;\mathrm{plaques}}{\mathrm{dilution}\mathrm{factor}\;\cdot \mathrm{phage}\mathrm{volume}\mathrm{added}}$$

### Denaturing Polyacrylamide Gel

An 8 m urea 10% polyacrylamide gel was prepared in a Tris/Borate/EDTA (TBE) buffer system. 18 µL of quenched cleavage reactions were heated at 90 °C for 1 min, cooled at ambient temperature for 5 min, and applied to the gel. Gels were run for a sufficient time to resolve cleavage products of EC1 from the full‐length sequence. Using the Cy2 filter set on a Cytiva Typhoon biomolecular imager (Center for Microbial Chemical Biology, McMaster University), electrophoresed gels were visualized by fluorescence scan. Image files were analyzed using ImageJ.^[^ ^41^ ^]^

### Polymerization

A modified version of the polymerization procedure described by Lin et al was used.^[^ ^25^ ^]^ Two separate solutions each containing acrylamide monomer and one of the acrydite modified oligonucleotide sequences were heated to 45 °C. Free radical polymerization was induced with the addition of APS and TEMED, at which point the tubes containing the solutions were put in a vacuum desiccator for 30 min. Final reagent concentrations are available in Table S2 (Supporting Information).

### Gold Nanoparticle Addition

A total of 8 mL of the BSA‐coated gold nanoparticles in DI water were centrifuged at 14 000 RPM for 15 min and the supernatant was removed. 40 uL of this remaining solution was added to each tube of polyacrylamide‐DNA solution to give them a red color, as well as 160 uL of DI water which was left for 24 h and then thoroughly mixed.

### Gel Fabrication

A total of 0.2 mL PCR tubes were placed in a heat block at 95 °C. A total of 3 uL of each of the two polyacrylamide‐DNA solutions were added to all of the PCR tubes, for a total volume of 6 uL. The complementary parts of the oligonucleotides result in DNA crosslinking of the polyacrylamide chains, causing gelation. The resulting hydrogels were then gradually cooled to room temperature in a PCR machine over 30 min, and the condensation settled at 8500 RPM using a VWR mini‐centrifuge. Smaller 2 uL hydrogels were then taken by pipette from these larger gels and used for all experiments described, excluding the optimization experiments for which the full 6 uL gels were used.

### Gel Optimization

*E. coli* K12 contaminated water, T7 bacteriophage, and MgCl_2_ buffer were added to each hydrogel and left to incubate for 2 days at 40 °C and 180 RPM. Gels were imaged and quantified as described in the following section.

### Gel Sensitivity Testing

A total of 15 uL of MgCl_2_ buffer was added to each hydrogel 24 h before sensitivity testing. Resuspended *E. coli* K12 was serially diluted in DI water to concentrations from 10^1^ to 10^7^ CFU mL^−1^. 70 uL of these dilutions were added to each gel, along with 15 uL of an autolyzed yeast extract solution at a concentration of 50 mg mL^−1^. The tubes containing the hydrogel, buffer, yeast extract, and bacteria were then incubated at 40 °C and 180 RPM. After 6 h 70 uL of T7 bacteriophage was added, and after 18 h the samples were removed from the shaker and imaged on an Epson Perfection V850 Pro scanner. Results were quantified by measuring the mean brightness of a consistent area of each tube (50 pixels in height and 100 pixels in width) with ImageJ software.

### Gel Specificity Testing

*E. coli* O157:H7, *A. lwoffii*, *B. subtilis*, *L. monocytogenes*, *P. aeruginosa*, and methicillin‐resistant *S. aureus* were serially diluted in DI water to concentrations of 10^3^ and 10^6^ CFU mL^−1^ and tested under the same conditions as the sensitivity experiment.

### Gel Stability Assessment

Prepared gels were stored in a sealed container at −20, 4, and 20 °C for 2 weeks, corresponding to storage in the freezer, fridge, and at room temperature. After storage, the gels were tested according to the normal procedure with *E. coli* K12 concentrations of 10^3^ and 10^6^ CFU mL^−1^ and then imaged. The 30 °C condition gels were stored for 8 days, with 3 uL of the MgCl_2_ buffer already on each gel to prevent them from drying out. For long‐term stability testing 6 uL gels were stored in the freezer at −20 °C for 5 months, before being pipetted into smaller 2 uL hydrogels and tested according to the normal procedure with spiked lake water samples.

### Environmental Samples

Lake water was collected in knee‐depth water from Lake Erie near Dunnville, Ontario. Cistern water was collected from a cistern on a rural property also in Dunnville, Ontario.

### Urine Testing

Urine samples were acquired from Hamilton General Hospital in Hamilton Ontario, Canada. Samples were collected according to the McMaster Research Ethics Board, with informed written consent. The REB number is 2062. Of these 12 clinical samples, 7 were positive for *E. coli* (>10^5^ CFU mL^−1^) and 5 were negative (Table S4, Supporting Information). Urine samples were mixed with DI water at a ratio of 1:99, and then tested with the platform following the procedure described in the “Gel Sensitivity Testing” methods section. For the spiked urine experiment, the *E. coli* negative clinical sample listed as sample 12 in Table S4 (Supporting Information) was spiked with *E. coli* to a concentration of 10^5^ CFU mL^−1^.

### Freeze Drying and SEM

DNAzyme hydrogels were incubated overnight at −80 °C, then freeze‐dried for 24 h at −51 °C under 0.11 mBar vacuum. Following freeze drying, the gels were attached to the SEM stub with double‐sided carbon tape. After being coated with 15 nm gold (Quorum 300T D Plus sputter coater) the samples were imaged on the JEOL6610 LV SEM. 200X and 1000X magnification images were taken with the secondary electron imaging (SEI) technique. Voltage was 5 kV. This took place at McMaster University (Ontario, Canada) at the Canadian Centre for Electron Microscopy (CCEM).

### Rheology

Rheology Peltier setup parameters were used as follows: cone‐plate geometry with 20 mm diameter, 1° cone angle, truncation gap 45 µm, sample amount 70 µL, test temperature 25 °C. Mineral oil was applied around the circumference of the plates to prevent evaporation. A time sweep was carried out for 3 min at a fixed strain of 1% and frequency of 1 Hz. The strain sweep was completed from 0.01 to 10 000% strain at a fixed frequency of 1 Hz, 10 points per decade.

### Artificial Intelligence Image Analysis

MATLAB's MathWorks Deep Learning Toolbox was used to train and test the CNN model. CNNs contain alternating convolutional and pooling layers, as well as a fully connected layer that decides the output according to the calculated probability of an image belonging to each output class (in this case, being positive or negative for *E. coli*). 88 images were used with a 70/30 training to validation split. Images were cropped to a consistent size of 1000 by 400 pixels with one tube in each image. The model was trained and validated over 30 iterations, ending with a final validation accuracy of 100%. Training accuracy/loss plots are shown in Figure S10 (Supporting Information).

### Statistical Analysis

In the graphs of Figures 3 and 4, quantified brightness values have been normalized to the mean brightness of their respective controls. Error bars on graphs represent the standard error of the mean. The number of samples for each experiment is specified in figure captions, and the minimum sample size used in this study is three. Ordinary one‐way ANOVA with Dunnett's multiple comparisons test was used to compare three or more groups, while two‐tailed t‐tests were used when comparing only two groups. Asterisks represent significant differences at corresponding significance levels of “ns” (*p* > 0.05), ^*^ (*p* ≤ 0.05), ^**^ (*p* ≤ 0.01), ^***^ (*p* ≤ 0.001), and ^****^ (*p* ≤ 0.0001). GraphPad Prism version 9.5.1 software was used for statistical analysis and graphs.

## Conflict of Interest

The authors declare no conflict of interest.

## Supporting information



Supporting Information

## Data Availability

The data that support the findings of this study are available from the corresponding author upon reasonable request.
