# Supplementary material for: Bacteriophage‐Activated DNAzyme Hydrogels Combined with Machine Learning Enable Point‐of‐Use Colorimetric Detection of Escherichia coli
Source: Adv Mater. 2024 Nov 26;37(3):2411173. doi: 10.1002/adma.202411173 (PMC11756048; doi:10.1002/adma.202411173)
Supplement: Supplementary file 1 — Supporting Information [file ADMA-37-2411173-s001.docx]

Supporting Information

**Bacteriophage-activated DNAzyme Hydrogels Combined with Machine Learning Enable Point-of-Use Colorimetric Detection of *Escherichia coli***

Hannah Mann^a^, Shadman Khan^b^, Akansha Prasad^b^, Fereshteh Bayat^b^, Jimmy Gu^c^, Kyle Jackson^a^, Yingfu Li^*c^, Zeinab Hosseinidoust^*a^, Tohid F. Didar^*b,d^, Carlos D. M. Filipe^*a^

^a^ Department of Chemical Engineering, McMaster University, 1280 Main Street West, Hamilton, ON L8S 4L8, Canada.

^b^ School of Biomedical Engineering, McMaster University, 1280 Main Street West, Hamilton, ON L8S 4L8, Canada.

^c^ Department of Biochemistry and Biomedical Sciences, McMaster University, 1280 Main Street West, Hamilton, ON L8S 4L8, Canada.

^d^ Department of Mechanical Engineering, McMaster University, Hamilton, ON L8S 4L7, Canada.

^*^To whom correspondence should be addressed. E-mail: filipec@mcmaster.ca, liying@mcmaster.ca, doust@mcmaster.ca, didart@mcmaster.ca

**Table S1.** DNAzyme and substrate sequences with acrydite modifications.

| **Component** | **Sequence** |
| --- | --- |
| DNAzyme | /5Acryd/GA TGT GCG TCT TGA TCG AGA CCT GCG ACA GGA AG |
| Substrate | /5Acryd/TT TTT ACT CTT CCT AGC TrAT GGT TCG ATC AAG A |


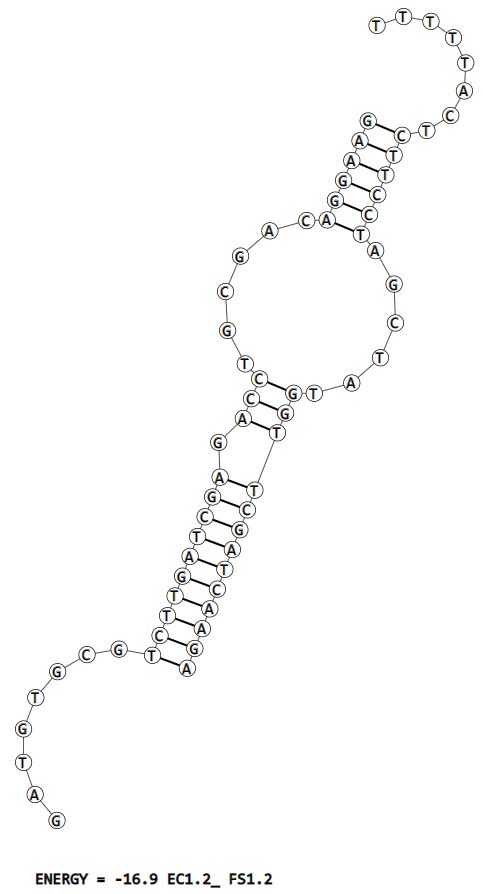


**Figure S1.** DNAzyme construct, shown without acrydite modifications.


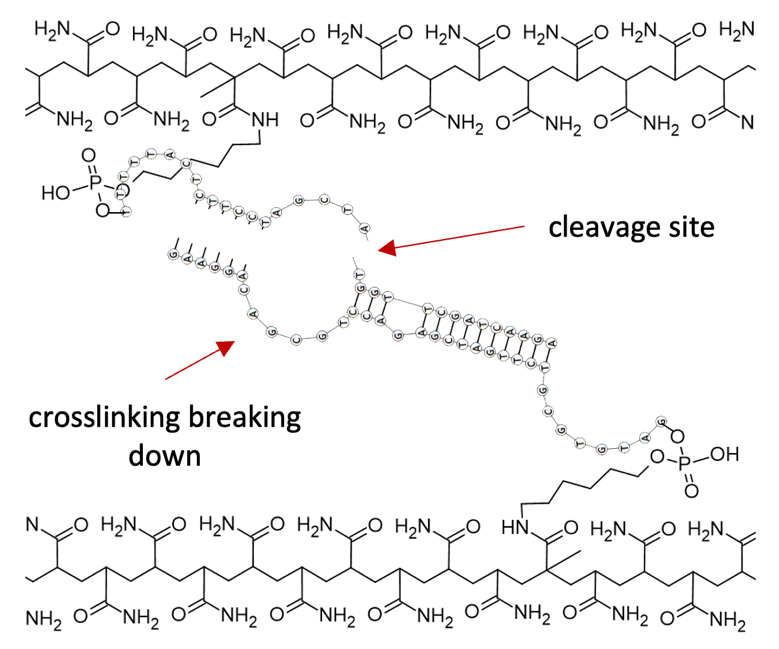


**Figure S2.** 2D chemical structure of the polymeric matrix showing an acrydite DNAzyme/substrate crosslink breaking down at its cleavage site.


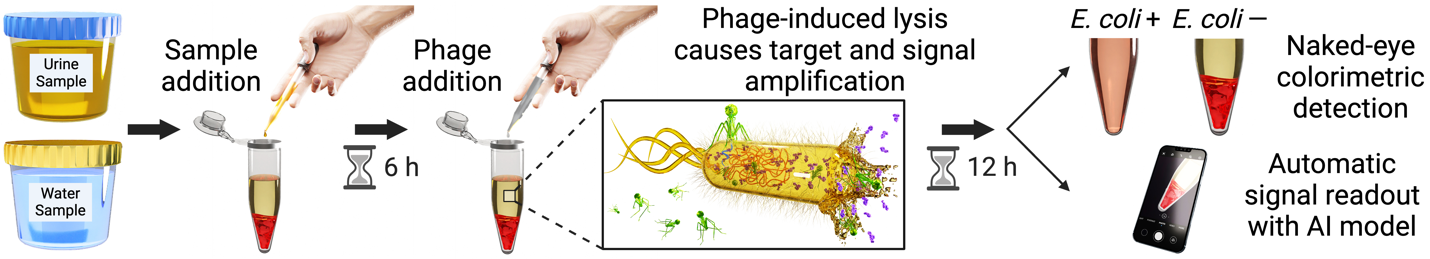


**Figure S3.** Schematic demonstrating the complete process of using the gel sensor platform.


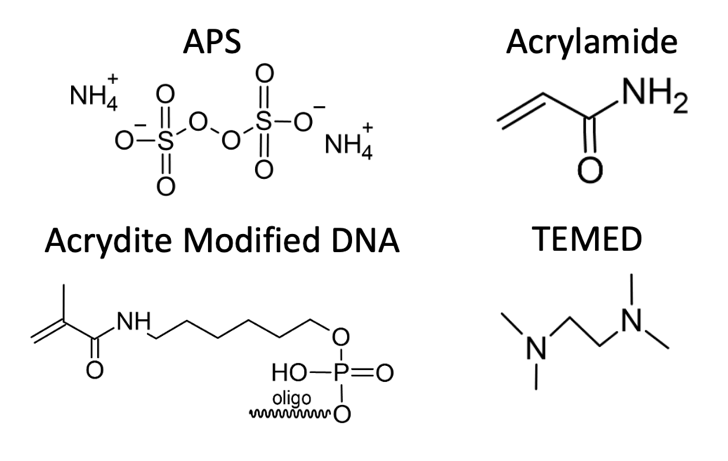


**Figure S4.** Chemical structures of reagents involved in the FRP reaction.

**Table S2.** Polymerization reagent concentrations and quantities.

| **Reagent** | **Concentration** | **Volume (µL)** |
| --- | --- | --- |
| Acrylamide | 25% in water (w/v) | 50 |
| DNA (DNAzyme or Substrate) | 3 mM | 16 |
| Water | N/A | 50 |
| APS | 10% in water (w/v) | 1.68 |
| TEMED | 10% in water (v/v) | 1.68 |


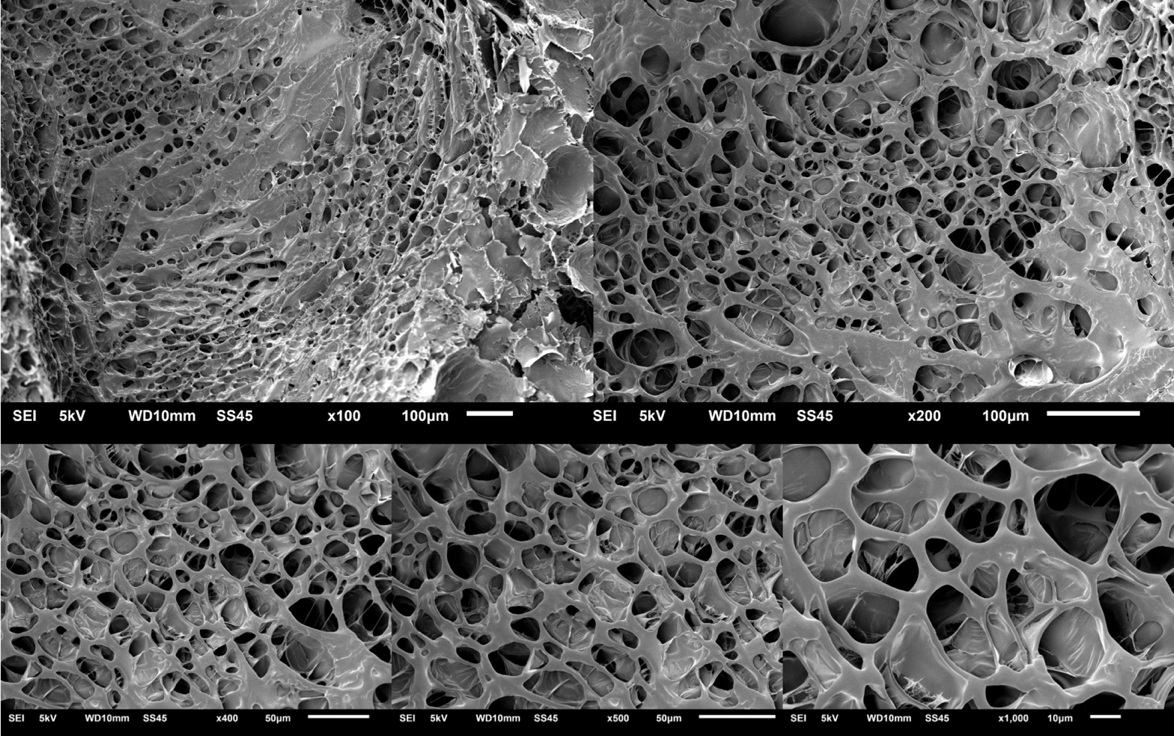


**Figure S5.** SEM Images of hydrogel at different magnifications.


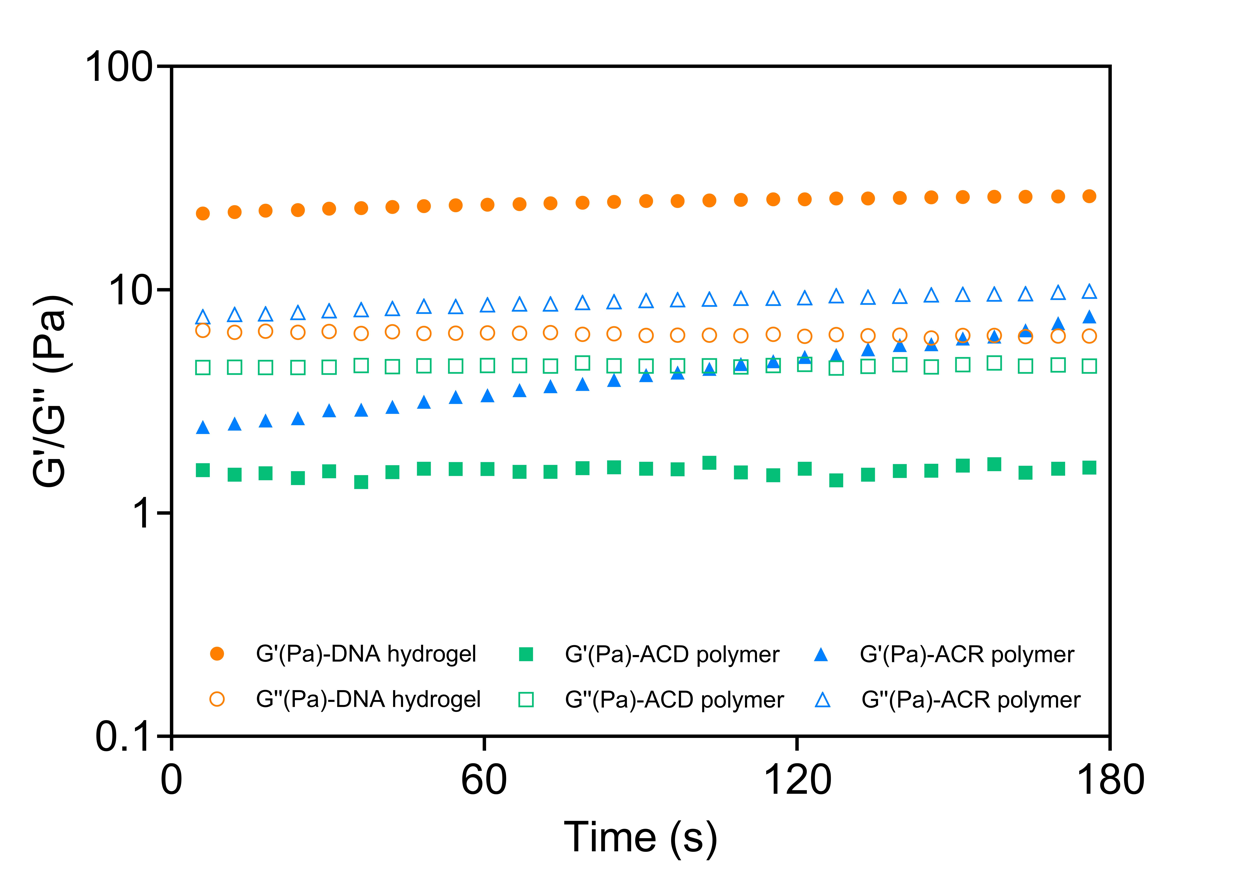


**Figure S6.** Rheology time sweep comparison of each sequence and the formed hydrogel. ACD refers to the polymer with the DNAzyme oligo, while ACR refers to the polymer with the substrate oligo.


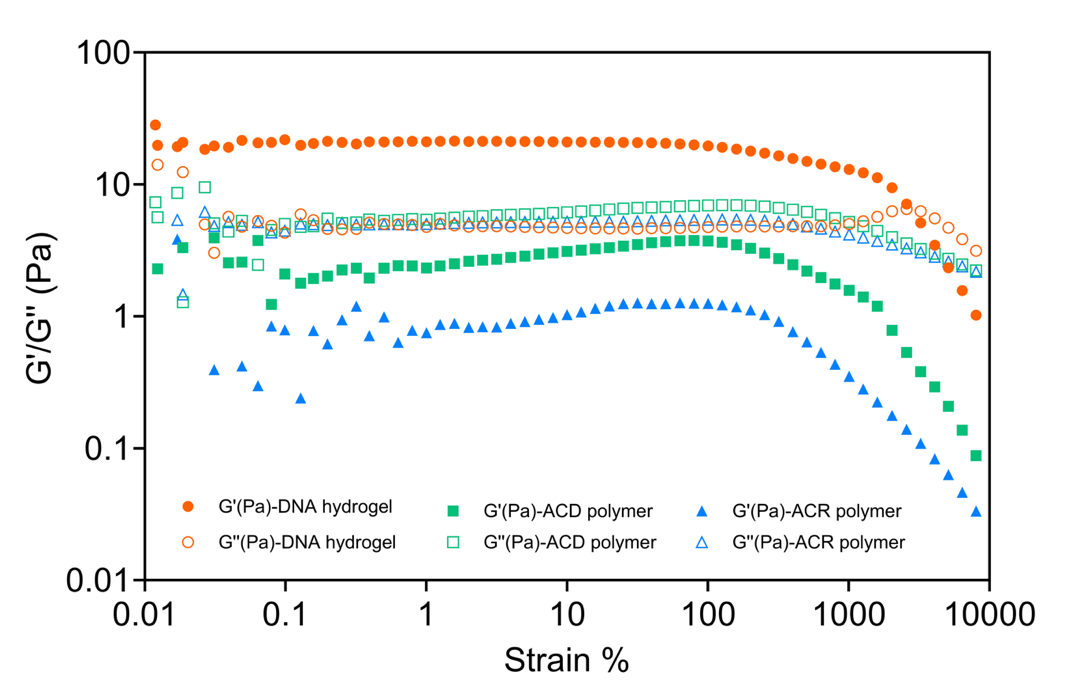


**Figure S7.** Rheology strain sweep comparison of each sequence and the formed hydrogel. ACD refers to the polymer with the DNAzyme oligo, while ACR refers to the polymer with the substrate oligo.


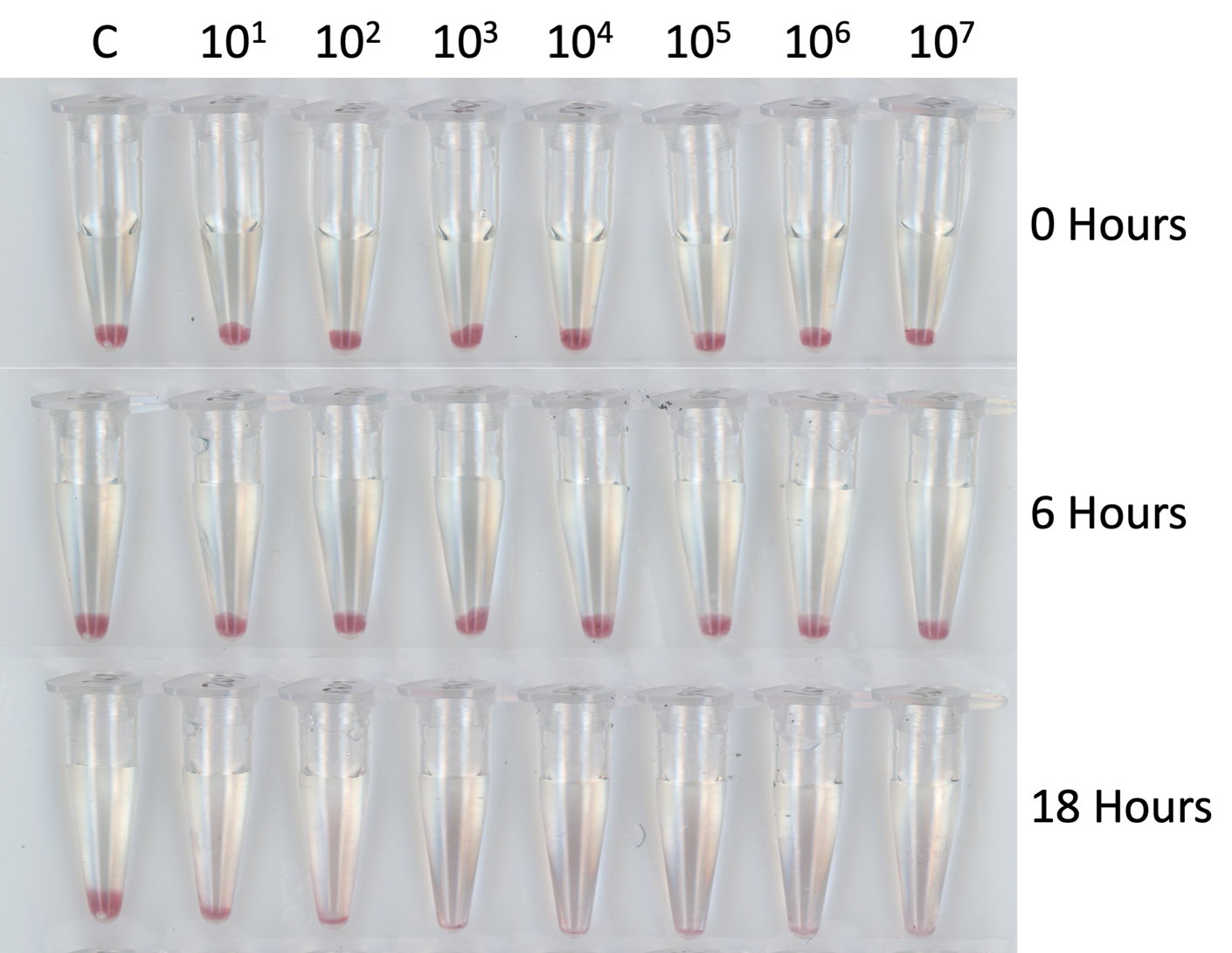


**Figure S8.** Sample optical images of hydrogel at several time points.

**Table S3.** pH measurements of water sources used for experiments.

| **Water Type** | **Reading 1** | **Reading 2** | **Reading 3** | **Average** |
| --- | --- | --- | --- | --- |
| Ultrapure Water | 7.60 | 7.27 | 7.29 | 7.39 |
| Lake Water | 8.01 | 8.05 | 8.07 | 8.04 |
| Cistern Water | 8.25 | 8.24 | 8.24 | 8.24 |


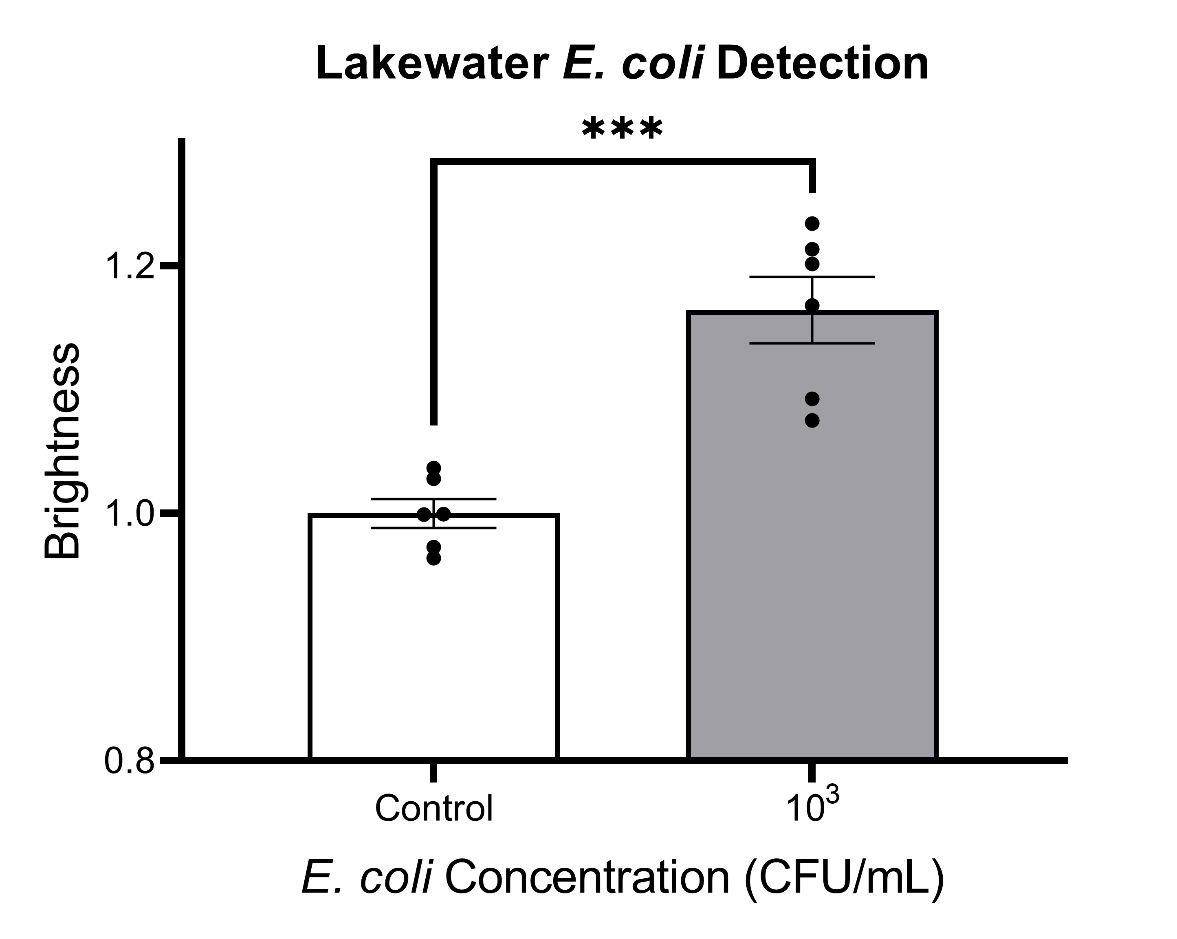


**Figure S9.** Detection performance of the platform for 10^3^ CFU mL^-1^ *E. coli* K12 with lake water samples. Sample size n = 6 for each concentration.


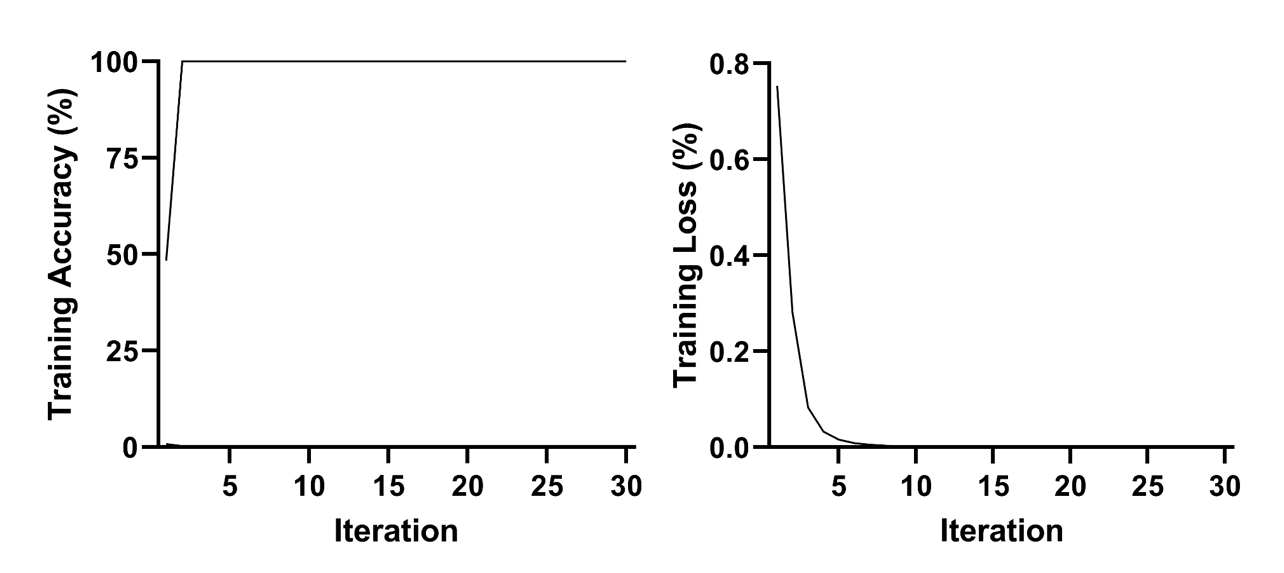


**Figure S10.** Training accuracy/loss plots from the CNN AI model.


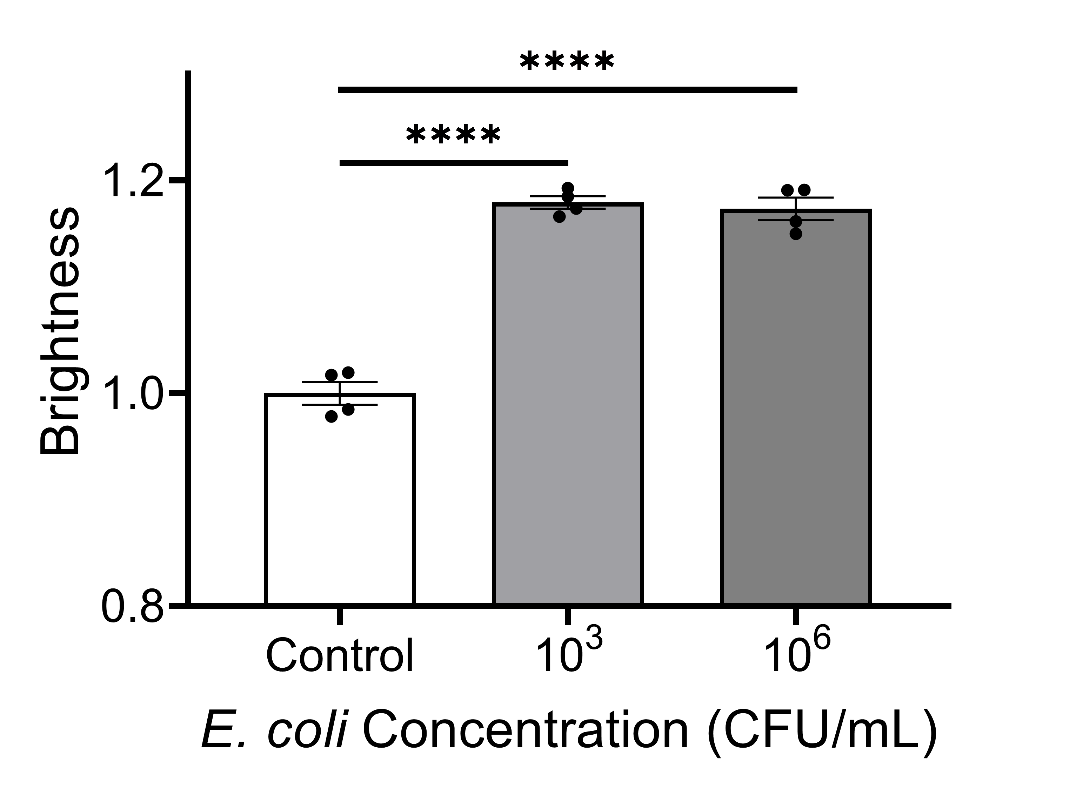


**Figure S11.** Detection performance of the platform after gels were stored for 8 days at 30 ℃, topped with 3uL of MgCl_2_ buffer to offset evaporation. Tested with 10^3^ and 10^6^ CFU mL^-1^ *E. coli* K12 in lake water samples. Sample size n = 4 for each concentration.


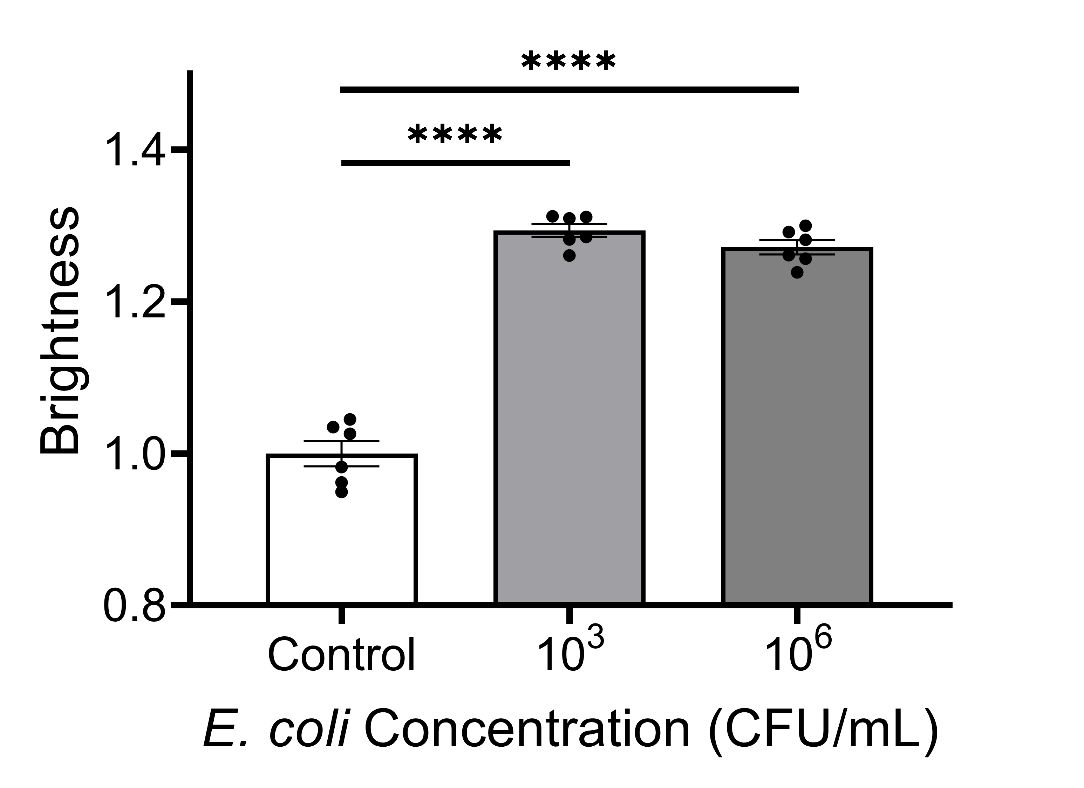


**Figure S12.** Detection performance of the platform after gels were stored for 5 months at -20 ℃ in the freezer. Tested with 10^3^ and 10^6^ CFU mL^-1^ *E. coli* K12 in lake water samples. Sample size n = 6 for each concentration.

**Table S4.** pH and infection status of clinical urine samples.

| **Sample Number** | **UTI Status** | **Sample pH** |
| --- | --- | --- |
| 1 | *E. coli* Infection (>10^5^ CFU/mL) | 5.26 |
| 2 | *E. coli* Infection (>10^5^ CFU/mL) | 5.10 |
| 3 | *E. coli* Infection (>10^5^ CFU/mL) | 6.49 |
| 4 | *E. coli* Infection (>10^5^ CFU/mL) | 4.91 |
| 5 | *E. coli* Infection (>10^5^ CFU/mL) | 6.07 |
| 6 | *E. coli* Infection (>10^5^ CFU/mL) | 6.21 |
| 7 | *E. coli* Infection (>10^5^ CFU/mL) | 5.79 |
| 8 | No Infection | 5.53 |
| 9 | No Infection | 6.73 |
| 10 | No Infection | 5.00 |
| 11 | No Infection | 6.11 |
| 12 | No Infection | 6.89 |


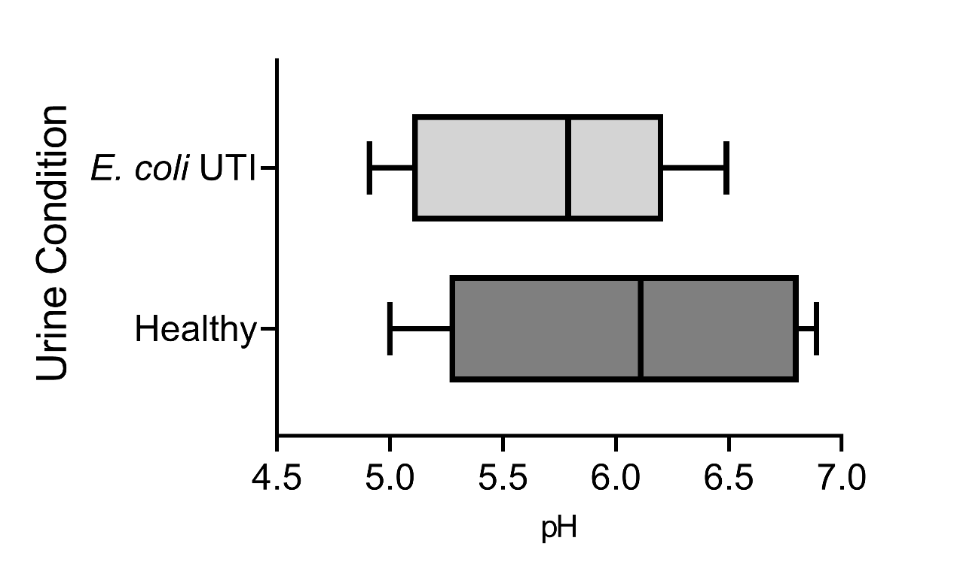


**Figure S13.** Box and whisker plot of the pH of clinical urine samples used, categorized by UTI status.
